# Simultaneous Automatic Electrochemical Detection of Zinc, Cadmium, Copper and Lead Ions in Environmental Samples Using a Thin-Film Mercury Electrode and an Artificial Neural Network

**DOI:** 10.3390/s150100592

**Published:** 2014-12-30

**Authors:** Jiri Kudr, Hoai Viet Nguyen, Jaromir Gumulec, Lukas Nejdl, Iva Blazkova, Branislav Ruttkay-Nedecky, David Hynek, Jindrich Kynicky, Vojtech Adam, Rene Kizek

**Affiliations:** 1 Department of Chemistry and Biochemistry, Faculty of Agronomy, Mendel University in Brno, Zemedelska 1, CZ-613 00 Brno, Czech Republic; E-Mails: george.kudr@centrum.cz (J.K.); nguyenviethoai@hus.edu.vn (H.V.N.); lukasnejdl@gmail.com (L.N.); iva.blazkova@seznam.cz (I.B.); brano.ruttkay@seznam.cz (B.R.-N.); d.hynek@email.cz (D.H.); vojtech.adam@mendelu.cz (V.A.); 2 Central European Institute of Technology, Brno University of Technology, Technicka 3058/10, CZ-616 00 Brno, Czech Republic; E-Mail: j.gumulec@gmail.com; 3 Karel Englis College, Sujanovo nam. 356/1, Brno CZ-602 00, Czech Republic; E-Mail: jindrak@email.cz

**Keywords:** automation, electrochemical detection, artificial neuronal network, robotic device, metal ions, environmental analysis

## Abstract

In this study a device for automatic electrochemical analysis was designed. A three electrodes detection system was attached to a positioning device, which enabled us to move the electrode system from one well to another of a microtitre plate. Disposable carbon tip electrodes were used for Cd(II), Cu(II) and Pb(II) ion quantification, while Zn(II) did not give signal in this electrode configuration. In order to detect all mentioned heavy metals simultaneously, thin-film mercury electrodes (TFME) were fabricated by electrodeposition of mercury on the surface of carbon tips. In comparison with bare electrodes the TMFEs had lower detection limits and better sensitivity. In addition to pure aqueous heavy metal solutions, the assay was also performed on mineralized rock samples, artificial blood plasma samples and samples of chicken embryo organs treated with cadmium. An artificial neural network was created to evaluate the concentrations of the mentioned heavy metals correctly in mixture samples and an excellent fit was observed (*R*^2^ = 0.9933).

## 1. Introduction

Metals mainly occur in the Earth's crust, however, urbanization and industrialization lead to their releasing into the biosphere, where they have become part of the air, soil, water and biota [1–3]. As a consequence of the metabolic similarity of toxic metals with non-toxic elements, they bind to the sulfhydryl groups of proteins causing negative effects, including mutagenesis [4,5]. Some metals, such as copper (Cu) and zinc (Zn), are essential micronutrients, although they are also toxic in higher concentrations. On the other hand, other metals as cadmium, lead and mercury can damage numerous biochemical pathways, even at low concentration. Due to this fact and the fact that the one-half of the World's population lives now in urbanized areas, metals continue to present a serious issue for public health [6].

Several methods have been developed to detect trace amounts of heavy metals. Conventional heavy metal detection methods include atomic absorption spectrometry (AAS), inductively coupled plasma mass spectrometry (ICP-MS) or inductively coupled plasma optical emission spectroscopy (ICP-OES) [7–9]. However, they do not meet the demands for portable, easy-to-use, quick and cheap analysis. In this field, electrochemical analysis of heavy metals is an alternative to conventional methods and provides a few attractive properties, in addition to a high degree of sensitivity [10–12]. Electrochemistry offers unique application possibilities in the field of heavy metal analysis due to the compact, simple and portable instrumentation, electrode miniaturisation and easy electrode modification [13–15]. Electrochemistry was also proved to be suitable method to analyse heavy metal contents in biological and environmental samples like body fluids, tissues or rocks [16–18]. *Gallus domesticus* hen and its embryos are broadly used as a model organism [19], where the liver represents the organ of initial Cd accumulation and recent study shows that high levels of Cd can be presented also in brain [20].

Among other electrode materials suitable for these purposes, mercury and carbon have been used the most frequently. In spite of the fact that mercury has unique physico-chemical properties and are widely used in trace heavy metal analysis, neither the dropping mercury electrode nor the hanging mercury drop electrode are suitable for automated analysis methods because of their mechanical instability (the mercury drop is easily dislodged). High consumption of metallic mercury in these cases also does not correspond with current trends in heavy metal analysis, since much attention has been focused on the use of more eco-friendly solid materials like carbon and chemical modification of its surface to improve sensitivity [21,22]. Thin-film mercury electrodes (TFMEs) are able to resolve these limitations [23]. They decrease mercury consumption, enable electrode manipulation and preserve the key mercury electrode properties like a wide cathodic potential window. In this case, mercury is electrodeposited from a solution with low mercury concentration on the surface of a solid electrode, which serves as a mechanical support and enables easier manipulation. In the case of heavy metals analysis, anodic stripping voltammetry (ASV) exhibits remarkable sensitivity [24,25]. A more negative potential than the standard redox potential of the heavy metal ions is used to preconcentrate them on the mercury surface. A subsequent linear increase of the potential stripps them back into the solution represented by increased current at a specific potential. Pulse techniques like differential pulse or square wave voltammetry suppressing the background current were successfully combined with ASV to lower the limits of detection [26]. The only alternatives to mercury for stripping techniques are Bi-modified electrodes, however, their limitations are low anodic potential [27].

Moreover, there are also demands on high throughput analysis in the field of environmental monitoring. The long-term monitoring of heavy metal pollution is the only way to meet national and international legislative measures implemented to decrease the anthropogenic pressure on the environment. The automatic handling of samples shortens time-consuming analysis, enables one to perform multiple analyses without continuous operator attention and is also consistent with current trends in analytical chemistry. Automatic flow-based voltammetric detections of heavy metals were proven to have good accuracy and reproducibility and succeeded in reducing analysis times [28,29]. Use of titre plates for automatic analysis enables one to avoid the need for complex microfluidics and electrochemical flow-cells [30]. The potential of the electrochemical robotic system for automated quantification of Ni^2+^ ion released from corroding nickel-titanium alloys was recently demonstrated [31]. The electrochemical robotic system was designed for automatically performing adsorptive stripping voltammetry in individual compartments of 24-well microtiter plates. Displacing the preloaded plates in the *x* and *y* directions, locating an electrode assembly into the solution in a selected well, conditioning the working electrode surface, and finally executing adsorptive stripping voltammetry are key actions that had to be automated for the sequential determination of Ni^2+^ concentrations in sample solutions in the different wells of the microtiter plate [31]. In another work, automatic ascorbic acid voltammetry was performed in 24-well microtiter plates. The automated assay used a movable assembly of a pencil rod working electrode, an Ag/AgCl reference electrode and a Pt counter electrode using differential pulse anodic stripping voltammetry (DPASV) for concentration-dependent current generation [30].

Data obtained by voltammetric sensors from multicomponent environments produces complex signals. To solve this problem, several electrode surface functionalizations were developed to improve the electrode selectivity [12,32]. Alternatively, a multivariate signal processing tool can be used. Among others, artificial neural network (ANN) software-based techniques were developed to analyse complex data sets, and they excel in modelling and calibrating complex analytical signals [33]. There are many types of ANNs that vary mostly in the architecture or in the way they learn. It was proved that using ANN data analysis interference between target heavy metals ions and the effect of sample matrix can be counterbalanced and this also enables evaluate overlapped voltammograms [34–36]. This paper describes a novel application of the electrochemical robotic device that provides a convenient electrochemical 24-well microtiter plate assay for the automated quantification of multiple heavy metal samples containing Cd(II), Cu(II), Pb(II) and Zn(II) in pure aqueous model solutions, mineralized rocks, chicken embryo liver and brain and human plasma samples spiked with metals. The choice of the carbon tip electrode modified with mercury film as a working electrode, signal stability and reproducibility of known metal solution levels are reported. Moreover, artificial neural networks software-based techniques developed to analyse the complex data sets were also used in this study [37–40], because it was found that the presence of copper and zinc in samples may lead to the formation of intermetallic compounds on the mercury film and this affects analysis of both elements [41]. The correct zinc concentration in the presence of copper and *vice versa* can be, however, determined by ANN processing [42,43].

## 2. Experimental Section

### 2.1. Chemicals

ACS purity (*i.e.*, chemicals meet the specifications of the American Chemical Society) sodium acetate trihydrate, acetic acid, Hg(NO_3_)_2_, water and other chemicals were purchased from Sigma-Aldrich (St. Louis, MO, USA) unless noted otherwise.

### 2.2. Instrumentation

An electrochemical robotic device (Sensolytics, Bochum, Germany) performed the automatic positioning of electrodes. Carbon tips (1 mL) were purchased from Tosoh Corporation (Tokyo, Japan) and were used as working electrodes after modification. Ag/AgCl/3M KCl as reference electrode (Metrohm, Herisau, Switzerland) and platinum wire (Metrohm, Herisau, Switzerland) as counter electrode were used. Electrochemical signals were recorded using a PGSTAT 101 potentiostat (Metrohm) and the NOVA 1.8 software (Metrohm) was employed for data evaluation. The electrode holder was printed by a PROFI 3D MARKER printing system (3Dfactories, Straznice, Czech Republic). Samples were measured in flat bottomed TPP tissue culture 24-well plates (Sigma-Aldrich).

#### 2.2.1. Electrochemical Robotic Device

Electrodes were placed into the holder fabricated using the 3D printer. The electrochemical robotic device (Sensolytics) positioning the electrodes included three motorized units ST4118M1804 (Nanotec, Munich, Germany) and positioning system (OWIS, Staufen, Germany). The first unit was rigidly connected to the vertical frame of the electrochemical robot. The electrode holder was attached to it and this enabled us to perform precise vertical (*z*) positioning of the electrode holder (up and down). The microtiter plate was placed on a horizontally (*x*/*y*) positioned board. Coordinates and the precise time of the holder and plate motion were controlled by the ELChemRo software (Sensolytics). We used the advanced settings of NOVA to prepare a script enabling us to set up the sequence of differential pulse voltammetric measurements with adjustable time intervals between individual measurements.

#### 2.2.2. Working Electrode

Automatic electrochemical detection was performed using a three electrodes system. A pipette tip made from polymeric material and coated by graphite enabled us to use it as a working electrode due to its conductive resin. Based on the mentioned facts, these electrodes can be used for detection of substances undergoing reduction and/or oxidation on the surface of such electrodes. In this study, detection of Cd(II), Pb(II), and Cu(II) was carried out by a bare working electrode. Carbon tip electrode modified with mercury film was employed for detection of Zn(II) ions (no reduction was observed using the bare electrode) and for detection of metal mixtures.

#### 2.2.3. Modification of Carbon Tips

The carbon tips were inserted into 0.01 M Hg(NO_3_)_2_ solution, prepared by the dissolution of 0.086 g mecury(II) nitrate in 25 mL of acidified (5% HNO_3_, *v*/*v*) Milli-Q water. A −0.9 V potential was applied to the electrodes for 60 s, which resulted in the formation of a thin-film of mercury on the surface of the working electrode [44].

#### 2.2.4. Method

We used differential pulse voltammetry for all measurements and measurement parameters were as follows: deposition potential −1.6 V, initial potential −1.6 V, end potential 0.1 V, step potential 0.005 (scan rate 50 mV·s^−1^), modulation amplitude 0.1 V, modulation time 0.004 s, interval time 0.1 s. All experiments were carried out at room temperature. Acetate buffer (0.2 M CH_3_COOH and 0.2 M CH_3_COONa) was used as the supporting electrolyte. The limit of detection was calculated as LOD = (3.3 × SD)/S, where *SD* = standard deviation of the response and *S* = slope of the calibration curve.

### 2.3. Statistical Analysis

First, simple regression was performed for each metal peak value–metal concentration pair. The following functions were tested: linear, logarithmic and exponential. The correlation of each regression was tested and then the optimal function for each metal was used. Based on these results, a nonlinear estimation using a user-determined regression function was created and the goodness of fit of the model was tested again. In the third step, an automated neuronal network was created. The following methods were tested: radial basis function and multilayer perceptron. The following activation functions were used for hidden and output neurons: identity, logistic, tan, and exponential. The number of hidden neurons was limited to 20 and was optimized during after the primary learning cycle. Weight decay was used to prevent overfitting using the following setting: 0.0001–0.001 (min–max) for both hidden and output layer. Data (645 samples in total) was randomly divided into a training group (70%), testing group (15%) and verification group (15%). A Broyden-Fletcher-Goldfarb-Shanno (BFGS) training algorithm was used. Unless noted otherwise, *p*-level 0.05 was considered significant. The software Statistica 12 (StatSoft, Tulsa, OK, USA) was used for analysis.

### 2.4. Sample Preparations

Fertilized egg of ISA brown hen (Integra, a.s., Zabcice, Czech Republic) was incubated in a RCom 50 MAX incubator (Gyeongnam, Changwon, Korea) at 37.5 °C and humidity control (45% rH). After 16 days of the incubation the embryo vitality was checked and then a solution of Cd(NO_3_)_2_•4H_2_O (4.5 mg·mL^−1^ in ACS water) was applied (500 μL) by injection using a Chirana T. injecta device (maximal volume: 1 mL, size: 0.33 × 12 mm) through a small hole in the egg shell into the air cell on the chorioallantoic membrane. After that the hole was covered by a plaster. The chicken embryo was incubated till the next day and then the brain and liver was extracted. From the chicken embryo, 10 mg of tissue (brain, liver) was equally removed, weighed and added to 500 μL of a mixture consisting of 350 μL 65% nitric acid (*v*/*v*) and 150 μL 30% hydrogen peroxide (*v*/*v*). The solutions were subjected to digestion in a microwave reaction system Anton Paar (Anton Paar GmbH, Graz, Austria) using the following conditions: time 40 min (10 min power 50, 30 min power 100 and 10 min power 0), 60 °C, Rotor-64MG5-16. Mineralized solutions (200 μL) were transferred to a 96-well Deepwell plate 96 evaporation plate (Eppendorf, Hamburg, Germany) and evaporated. For evaporation of samples an Ultravap 96 nitrogen blow-down evaporator with spiral needles (Porvair Sciences, Leatherhead, UK) was used. Finally, the solutions were dissolved in 0.2 M acetate buffer (200 μL, pH 5.0) and were diluted 10-fold with the same acetate buffer prior to analysis. Rock samples (10 mg) were prepared in the same manner as chicken samples. For mineralization the following conditions were used: time 110 min (100 min power-100 and 10 min power-0), 100 °C, Rotor-64MG5-16 and samples were diluted 1000-fold. The plasma samples with random heavy metal concentrations (0–6 μg·mL^−1^) were prepared as follows: to 10 μL of human plasma specific amount of metals ion stock solutions were added. Mineralized and evaporated samples were diluted to the original volume, then they were diluted 10-fold with acetate buffer (0.2 M, pH 5.0) and used for ANN evaluation.

### 2.5. Determination of Cadmium by Atomic Absorption Spectrometry

Cadmium was also determined on an Agilent Technologies 80 Z atomic absorption spectrometer (Agilent, Santa Clara, CA, USA) with electrothermal atomization. The spectrometer was operated at the 228.8 nm resonance line with a spectral bandwidth of 0.5 nm. The sample volume (20 μL) was injected into the graphite tube. The flow of argon inert gas was 300 mL·min^−1^. Zeeman background correction was used with a field strength of 0.8 Tesla. The absorption signal was evaluated in peak height mode with seven point smoothing.

### 2.6. X-Ray Fluorescence Analysis (XRF)

The rock samples were measured on a Spectro Xepos apparatus (Spectro Analytical Instruments, Kleve, Germany) using an anode X-ray tube with Pd anode working at a voltage of 44.69 kV and a current of 0.55 mA. Signals were detected with Barkla scatter aluminium oxide for 300 s. For excitation three secondary targets (Mo, Al_2_O_3_ and high-ordered pyrolithic graphite crystal) were used. The excitation geometry was 90°. The crushed samples were measured through the PE bottle side wall 20 mm above the bottom. The Spectro Xepos software and TurboQuant method were applied for data analysis.

## 3. Results and Discussion

### 3.1. Automatic System for Heavy Metal Detection

**Figure 1.**
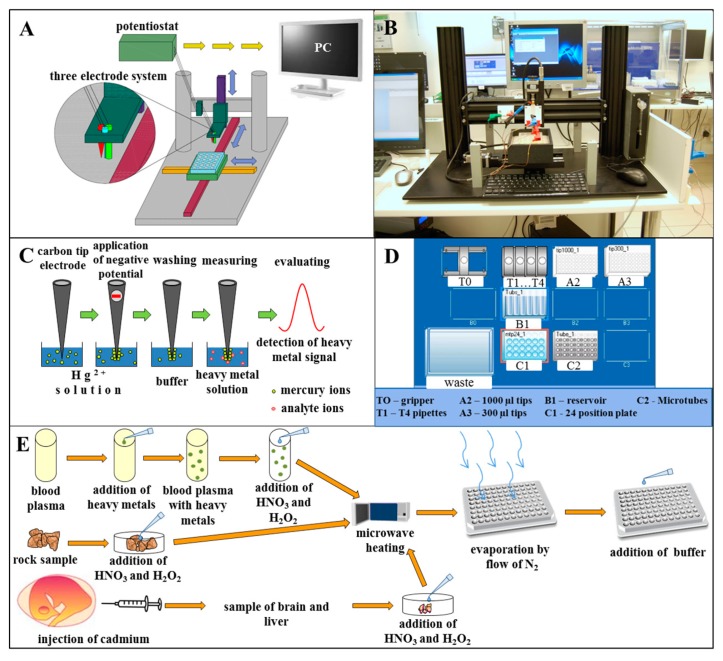
(**A**) Scheme and (**B**) photo of robotic device with electrochemical three-electrode detection system; (**C**) Individual steps of thin mercury film preparation on the surface of carbon tip electrode; (**D**) Scheme of automated pipetting system epMotion 5075 desktop, which was used to add buffer to mineralized samples of rocks; (**E**) The samples of blood plasma, stone and chicken embryo organs preparation prior to heavy metals detection.

Automation or semi-automation of analysis reduces time-consuming manual operations and costs. We used an electrochemical detection method (differential pulse voltammetry) with all its known advantages (easy-to-use, good sensitivity, cheap instrumentation) for automatic simultaneous detection of cadmium, zinc, copper and lead ions in various types of real samples. The whole system consisted of detection and positioning parts. Detection was performed using a classical three-electrode system (working, reference and auxiliary electrode). Electrodes were fitted to a movable holder and positioned using an electrochemical robotic device schematically depicted in Figure 1A and photographed in Figure 1B. Scripts were created to precisely control the timing of electrochemical measurements and movements of the electrode holder with the electrodes. At first, we used a disposable carbon tip as a working electrode, nevertheless this electrode was not able to detect zinc ions. Hence, we fabricated thin-film mercury electrodes (TFMEs) by electrodeposition of mercury (II) ions from solution onto the carbon surface of electrodes (Figure 1C) and its sensitivity was compared with a disposable carbon tip electrode. Due to the fact that we focused on automation, the electrochemical robotic system was used to perform plating automatically and the automated pipetting device was used to prepare different heavy metal concentrations for calibration curve measurement and mixing samples with buffer. It is well known that oxidation/reduction of some metals on the surface of TFME can be affected by the presence of other metals by forming of intermetallic compounds [45]. Therefore, we used statistical methods (linear regression, multiregression model and finally a neural network) to reduce measurement errors and evaluate the detected concentrations of target heavy metals [42,43]. Complex analytical signals of mixture samples obtained by arrays of potentiometric electrodes or by voltammetric systems (not those based on specific receptors) mostly require application of chemometric tools [46,47]. We decided to use an artificial neural network for these purposes since it is an effective instrument to analyse these types of multivariate signals and is able to recognise specific patterns in data sets. It can be considered as one of the most important tools in this kind of analysis and is not only broadly used in evaluation of redox, but also optical signals [48,49]. Gutés *et al.* emphasized the need for voltammetric signal pre-processing before ANN modelling in order to reduce ANN training time and create a more accurate network [50]. A wavelet transform was previously used to extract the most relevant information from voltammograms [51]. We used individual ions peak heights as the ANN input data, because Cd(II) and Zn(II) ions in samples tended to affect the peak heights of each other instead of overlapping (peaks of Cd(II), Cu(II) and Pb(II) and Zn(II) are well separated) [41]. The neural network reliability was tested using 22 mineralized blood plasma samples with random heavy metal concentrations (0.0–6 μg·mL^−1^) and 20 randomly selected heavy metal mixtures (0.01–8 μg·mL^−1^). The automatic electrochemical robotic device and neural network were also used for evaluation of heavy metal content in rocks and chicken embryo tissues exposed to cadmium(II) ions. Addition of buffer to mineralized rock samples, chicken brain and liver and artificial plasma samples was performed by an automated pipetting device (Figure 1D). The way samples were treated is shown in Figure 1E.

### 3.2. Optimization of the Automatic System for Heavy Metal Detection

The automatic system for heavy metal detection was optimized for determination of the four metal ions (Cd(II), Cu(II), Pb(II) and Zn(II)). The optimization was focused on monitoring of the electrochemical response of the individual elements depending on the increasing accumulation time within the range from 0 to 300 s. Longer accumulation times were not investigated because of our desire to shorten the analysis to a maximum of 5 min. Thereafter, calibration curves were determined and limits of detection (LODs) were calculated. For values resulting from the calibration curve double-sided reliability bands were created, what is the part of the plane limited by straight lines, where the observed calibration points fall within with 95% probability [52]. Further, the sensitivity of the WE before and after modification with mercury film was compared by plotting the slopes of the calibration curves in the column graph. Due to the fact that lower limits of detection were attained by the mercury-modified carbon tip, calibration curves were determined within a linear range of concentrations (0.6, 1.25, 2.5 and 10 μg·mL^−1^). This linear range is common for both unmodified and modified WE, and therefore all the slopes could be compared. Finally, the automatic system was verified by comparing the electrochemical results with atomic absorption spectrometry (AAS) data.

**Figure 2.**
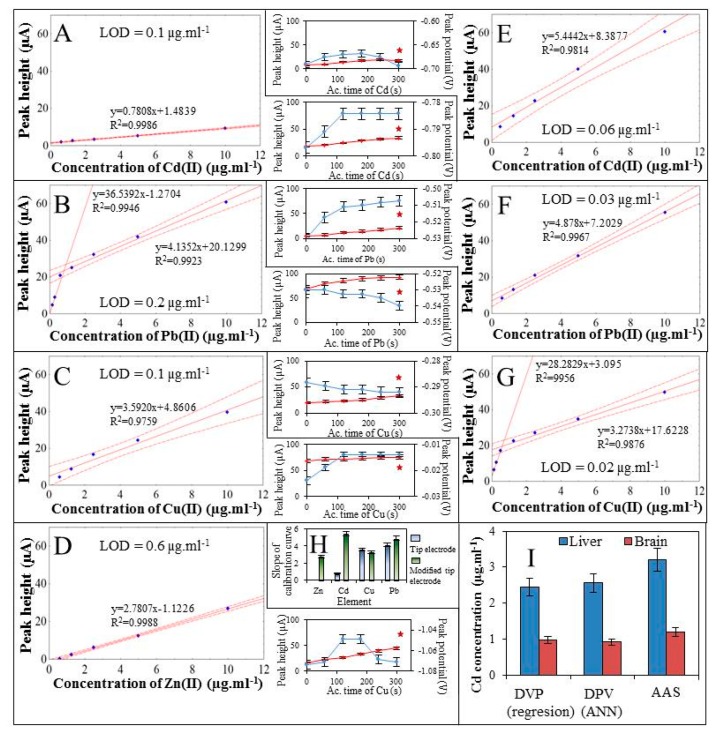
Calibration curves of (**A**) cadmium(II) (0.6–10 μg·mL^−1^); (**B**) lead(II) (0.16–10 μg·mL^−1^) and (**C**) copper(II) ions (0.6–10 μg·mL^−1^) measured using carbon tip electrode. Calibration curves of (**D**) zinc(II); (**E**) cadmium(II); (**F**) lead(II) (all 0.6–10 μg·mL^−1^) and (**G**) copper(II) ions (0.16–10 μg·mL^−1^) measured using thin film mercury electrode. (**H**) The graph of optimized time of accumulation (0–300 s) (**red line**) is connected with appropriate calibration curve and shows the changes of peak potential (**blue line**). Comparison of slopes of calibration curves measured using carbon tip electrode (blue bar) and thin film mercury electrode (**green bar**); (**I**) The amount of cadmium ions detected (by AAS, DPV and ANN) in brain and liver of chicken embryo (16 day) exposed to cadmium (II) ions (0.5 mg) by injection to air cell.

At first, the electrochemical responses of the studied metals on the unmodified carbon tip WE were studied. Cd(II), Cu(II), and Pb(II) ion electrochemical signals were measured, but Zn(II) ions did not give any signal at the bare electrode. For this reason, the electrochemical optimization was performed only for the detected ions. The best electrochemical response of metal ions was achieved when using an accumulation time of 300 s. This accumulation was applied for all measurements. For the analysis of Cd(II) ions the electrochemical signal gave its maximum at a potential of −0.69 V and LOD was estimated as 0.1 μg·mL^−1^ (Figure 2A). For the analysis of Pb(II) ions the peak maximum was detected at a potential of −0.51 V and LOD = 0.2 μg·mL^−1^ was estimated (Figure 2B). For the analysis of Cu(II) ions the peak maximum was detected at a potential of −0.29 V and LOD = 0.1 μg·mL^−1^ was found (Figure 2C).

The next step was to estimate the LOD of electrochemical determination of metal ions when using mercury film modified carbon tip as WE. It was found that due to the modification of WE with mercury film the electrochemical signal of Zn(II) was recorded. The signal of Zn(II) ions showed a maximum at a potential of −1.07 V and LOD was estimated as 0.6 μg·mL^−1^ (Figure 2D). For the analysis of Cd(II) ions the peak maximum was detected at a potential of −0.7 V and LOD as 0.06 μg·mL^−1^, which is 21 times less than when measured with the unmodified WE, was found (Figure 2E). For the analysis of Pb(II) ions the peak maximum was detected at a potential of −0.54 V and LOD as 0.03 μg·mL^−1^, which is five times less than when measured with the unmodified WE (Figure 2F). For the analysis of Cu(II) ions the peak maximum was detected at a potential of −0.29 V and LOD as 0.02 μg·mL^−1^, which is five times less than when measured with the unmodified WE (Figure 2G).

Further, for the use of the slopes of the calibration curves (Figures 2A–2G), sensitivities of the unmodified and modified WE for analysis of individual metal ions were compared (Figure 2H). It was found that modification of WE with mercury film had the greatest effect on detection of Zn(II) and Cd(II) ions. Detection of Zn(II) ions was possible only due to the WE modification. For detection of Cd(II) ions the sensitivity was increased 7-fold. On the other hand an increase in sensitivity was not demonstrated for the detection of Cu(II) and Pb(II) ions within a 5% error bar. Finally, brain and liver samples of the real-chicken embryo treated with Cd(II) ions were analysed. For these samples Cd(II) ions were determined. Comparison of the results measured by both DPASV (without stirring since the samples were placed in a titre plate) and AAS coincided within 10% error, as shown in Figure 2I.

### 3.3. Rock Analysis

**Figure 3.**
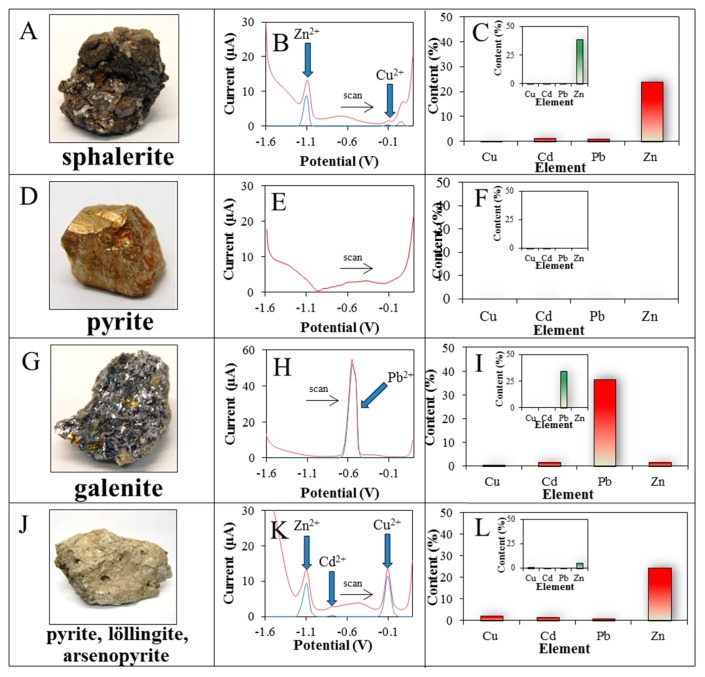
(**A**) Photo, original (red) and (**B**) baselined voltammogram (blue); (**C**) the element content in the rock calculated by neuronal network from voltammograms and element content measured using XRF (inserts) of rocks containing (**D**–**F**) sphalerite and pyrite; (**G**–**I**) galenite; and (**J**–**L**) arsenopyrite, pyrite, and löllingite.

Four different rocks were analysed electrochemically using TFME and the obtained results were compared with the X-ray fluorescence analysis (XRF) ones. Two rock samples consisting predominantly of the minerals sphalerite (ZnS) and pyrite (FeS_2_) were obtained in Madan in Croatia, and further galenite (PbS), and a mineral association of arsenopyrite (FeAsS) with pyrite and löllingite (FeAs_2_) were obtained in Panasqueira in Portugal (Figure 3).

For the first sample of analysed rock, which was composed predominantly of the mineral sphalerite (Figure 3A), the highest content of Zn(II) (24%) and traces of Cd(II) (1.5%), Pb(II) (1.0%) and Cu(II) (0.3%) were determined electrochemically and evaluated by ANN (Figure 3B). Similar results were obtained by XRF analysis (Figure 3C). In addition the elements Fe (10%) and S (12% of detected elements) were further detected (not shown).

For the second sample of analysed rock composed mainly of pyrite (Figure 3D), none of the analysed metals was determined electrochemically (Figure 3E and F). Using XRF analysis the elements Fe (47%), S (30%), Mg (1.22%) and Cu (1.35%) were detected (not shown), which corresponds essentially to the elemental composition of pyrite.

For the third sample of analysed rock, which was composed mainly of the mineral galenite (Figure 3G), the highest content of Pb(II) (37%) was determined electrochemically and this corresponded to the composition of this mineral (Figure 3H). Further, Si (8.5%) and minor amounts of Zn (1.8%), Cd (1.6%), and Cu (0.6%) ions were also determined (Figure 3I). Similar results were obtained by XRF analysis (Figure 3H and I).

For the fourth sample of analysed rocks, consisting primarily of the minerals arsenopyrite, pyrite and löllingite (Figure 3J) the largest amount of Zn(II) (25%) and in smaller amounts Cu(II) (2.0%), Cd(II) (1.4%) and Pb(II) (1.0%) were determined electrochemically (Figure 3K and L). Similar results were obtained using XRF analysis, but the elements Fe (32%), As (>18.29%), S (8%) and Sn (>2.875%) were also found (not shown).

### 3.4. Identification of Regression Function

A total of 645 mixture combinations of Zn(II), Cd(II), Cu(II), and Pb(II) standard concentrations within the range 0–10 μg/mL were prepared (appropriate amounts of the corresponding nitrates were dissolved in water). First, simple linear regression was performed to reveal the associations between peak values and concentrations using the following equation

(1) $$y_{zn}=a_{zn}+b_{zn}x_{zn}$$

where *y* indicates the concentration of each metal, *x* indicates the peak value for each metal and *a* and *b* are constants for each metal (Table 1). The goodness of fit of the model was as low as *R*^2^ = 0.87 for copper. Therefore, other functions were tested. Highest R squared (*R*^2^ = 0.92) was observed for the exponential function. Based on these results a multiple regression model using the following combined linear/exponential regression function was used:

(2) $$y_{zn}=b_{zn}x_{zn}+b_{cd}x_{cd}+b_{Pb}x_{Pb}+b_{cu}e^{\left(a_{cu}x_{cu}\right)}$$

where *y* indicates the concentration of each metal, x indicates the peak value for each metal, *b* is a constant (different for each metal), and *e* is the Euler constant (Table 1). The performance of this model was still weak for the calculation of copper concentration (R^2^ = 0.87). Therefore, instead of fitting another higher order or other complex functions, regression using automated neural networks was performed.

### 3.5. Building a Neural Network Model

Both the radial basic function and multilayer perceptron approaches were used for training with the following activation functions, which were used for hidden and output neurons: identity, logistic, tan, and exponential. In the initial training set total 10,000 training cycles were performed with weight decay and the five best were retained. The number of hidden neurons was limited to 20. After the initial training, the highest observed network performance was observed in a network with 19 hidden neurons, and exponential and logistic hidden and output activation functions, respectively. The goodness of fit of the model was 0.9996 for both test and validation (Table 2).

**Figure 4.**
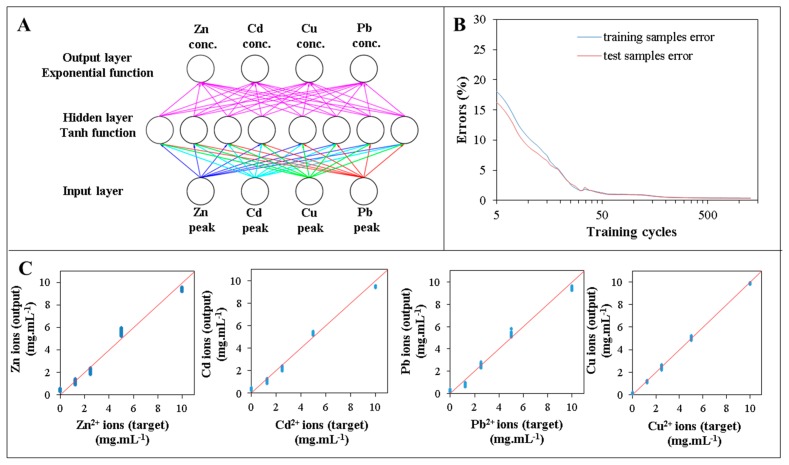
(**A**) Design of the final custom neuronal network model with four input, eight hidden and four output neurons. Note input layer neurons use identity functions; (**B**) Training of the network with the employment of the stopping conditions to prevent overfitting. Network was trained in the 1237th cycle, when the test error started to increase; (**C**) Testing the goodness of the fit of the target (known concentration) and output (neuronal network result).

Based on this network, the number of hidden neurons was optimized using a custom network design with enabled training stopping conditions. The number of hidden neurons was optimized in a range 0–30. The activation function for hidden and output layer was exponential and logistic with weight decay activated. When the number of hidden neurons increased from 1 to 8, the validation performance of the network increased significantly from 0.36 to 0.995. Subsequent increase in number of hidden neurons did not enhance the performance of the network significantly, therefore, eight hidden neurons was used as a model for final analyses (Table 2 and Figure 4A). Decreasing evaluation errors of training and testing samples were plotted against number of training cycles (Figure 4B).

Network name includes training method, and number of input-hidden-output neurons. MLP, multilayer perceptron. Training cycle indicates cycle number when the network was created (in the case of initial training) or the training was stopped by stopping conditions (in the case of final network design).

### 3.6. Measurements of a Blood Plasma and Unknown Samples

Consequently, a model was employed for the measurement of the 22 blood plasma samples, to which defined heavy metal concentrations were added (Table 3). The goodness of fit for the neuronal network was 0.995, 0.998, 0.993 and 9.999 for Zn, Cd, Pb, and Cu ions, respectively. Consequently, the model was tested on validation sample and goodness of fit was tested again. The R^2^ was as follows: 0.996, 0.998, 0.997, and 0.999 for Zn(II), Cd(II), Pb(II) and Cu(II), respectively. The results of these validations indicate significant improvement over using a general regression model with user defined function (compare the goodness of fit in Tables 1 and 2).

Concentration targets are concentrations added to blood plasma samples and prepared as custom concentrations for validation samples. Goodness of fit was tested separately for both sample sets. Correlations analysis of input heavy metals concentrations and output results were performed (Figure 4C).

## 4. Conclusions

In this work electrochemical system for automatic detection of heavy metals was developed. Using this system rock samples, blood plasma samples and organs of chicken embryos were successfully analysed. The accuracy of the system was verified by atomic absorption spectrometer (AAS) and X-Ray fluorescence (XRF). Furthermore, the different mathematical models were used to calculate the mutual interactions between the individual electrochemical signals in the multi element analysis. The performance of simple linear regression and multiple regression models (combination of linear and exponential regression) for determining copper concentrations correctly was weak. Based on this fact, an artificial neural network model was built and used for the correction of results in mixtures of metal samples. Perfect fit of this model was found (*R*^2^ = 0.9933).

## Figures and Tables

**Table 1.** Parameter estimates for simple regression and nonlinear estimation used in the optimization steps of the model. Parameters *a* and *b* for each metals are those used in the Equations (1) and (2). * indicate parameter is significant for calculation and therefore was used for the model.

**Model**	**Parameter**	**Parameter Estimates (95% Confidence Interval)**					

		**Zn(II)**	**Cd(II)**	**Pb(II)**	**Cu(II)**
Simple linear regression					

	a	* −0.56	* −0.62	* −0.52	* −1.47
	b	* 0.72	* 0.67	* 0.18	* 0.20
	model R^2^	0.98	0.98	0.98	0.87

Nonlinear estimation					

	b_Zn_	* 0.72 (0.70–0.73)	* 0.12 (0.11–0.13)	* −0.02 (−0.03–−0.01)	* 0.03 (0.00–0.05)
	b_Cd_	* 0.15 (0.13–0.16)	* 0.74 (0.73–0.76)	* −0.02 (−0.04–−0.01)	0.00 (−0.02–0.03)
	b_Pb_	0.00 (0.00–0.01)	0.00 (0.00–0.00)	* 0.19 (0.18–0.19)	0.00 (−0.01–0.00)
	b_Cu_	* −2.61 (−2.94–−2.28)	* −2.94 (−3.19–−2.7)	0.02 (−0.27–0.31)	* 0.46 (0.33–0.59)
	a_Cu_	0.00 (0.00–0.00)	0.00 (0.00–0.00)	0.00 (−0.31–0.31)	* 0.06 (0.05–0.06)
	model R^2^	0.98	0.98	0.98	0.87

**Table 2.** Results of the neuronal network learning optimization.

**Training Network Name**	**Network Performance (*R*^2^)**			**Network Error**			**Training Cycle**	**Activation Function**	
	
	**Training**	**Test**	**Validation**	**Training**	**Test**	**Validation**		**Hidden**	**Output**
Initial training									

MLP 4-17-4	0.9993	0.9993	0.9993	0.0362	0.0347	0.0367	210	Logistic	Exponential
MLP 4-19-4	0.9996	0.9996	0.9996	0.0261	0.0222	0.0291	699	Exponential	Logistic
MLP 4-19-4	0.9995	0.9995	0.9994	0.0292	0.0261	0.0320	232	Exponential	Logistic
MLP 4-14-4	0.9992	0.9990	0.9993	0.0 394	0.0461	0.0367	589	Tan	Identity
MLP 4-15-4	0.9993	0.9993	0.9993	0.0348	0.0333	0.0362	722	Tan	Exponential

Final network for further deployment									

MLP 4-8-4	0.9941	0.9924	0.9933	0.3744	0.3641	0.4275	1237	Exponential	Logistic

**Table 3.** Employment of the network on the measurement of the artificial blood plasma samples and set of validation samples.

**Group No. of Sample**	**Concentration Targets**				**Neuronal Network Outputs**			

	**Zn(II) (μg·mL^−1^)**	**Cd(II) (μg·mL^−1^)**	**Pb(II) (μg·mL^−1^)**	**Cu(II) (μg·mL^−1^)**	**Zn(II) (μg·mL^−1^)**	**Cd(II) (μg·mL^−1^)**	**Pb(II) (μg·mL^−1^)**	**Cu(II) (μg·mL^−1^)**
blood plasma samples measurement								

1	6.00	6.00	6.00	6.00	6.61	6.48	6.00	6.25
2	6.00	6.00	6.00	4.00	6.58	6.49	6.01	3.94
3	6.00	6.00	6.00	2.00	6.54	6.49	6.03	2.25
4	6.00	6.00	6.00	1.00	6.49	6.49	6.05	1.11
5	6.00	6.00	6.00	0.00	6.49	6.50	6.04	0.17
6	6.00	6.00	4.00	6.00	6.59	6.47	4.51	6.24
7	6.00	6.00	2.00	6.00	6.54	6.44	2.20	6.24
8	6.00	6.00	1.00	6.00	6.48	6.37	0.63	6.22
9	6.00	6.00	0.00	6.00	6.42	6.29	0.20	6.21
10	6.00	4.00	6.00	6.00	6.75	4.18	5.97	6.23
11	6.00	2.00	6.00	6.00	6.81	1.99	5.93	6.21
12	6.00	1.00	6.00	6.00	6.87	0.95	5.92	6.18
13	6.00	0.00	6.00	6.00	6.88	0.32	5.93	6.14
14	4.00	6.00	6.00	6.00	5.27	6.50	5.95	6.24
15	2.00	6.00	6.00	6.00	1.88	6.45	5.80	6.22
16	1.00	6.00	6.00	6.00	1.00	6.49	5.74	6.20
17	0.00	6.00	6.00	6.00	0.37	6.47	5.66	6.18
18	0.00	0.00	0.00	4.00	0.49	0.50	0.38	3.81
19	0.00	0.00	4.00	0.00	0.49	0.47	4.77	0.10
20	0.00	4.00	0.00	0.00	0.45	4.18	0.31	0.09
21	4.00	0.00	0.00	0.00	5.08	0.38	0.27	0.08
22	0.00	0.00	4.00	4.00	0.46	0.46	4.64	3.91
*R*^2^ of the network				0.995	0.998	0.993	0.999	

Validation samples											

1	8.00	8.00	8.00	8.00	8.42	8.59	8.53	9.02
2	8.00	8.00	8.00	4.00	8.38	8.60	8.52	3.82
3	8.00	8.00	8.00	2.00	8.35	8.61	8.53	2.11
4	8.00	8.00	8.00	1.00	8.31	8.61	8.54	1.04
5	8.00	8.00	8.00	0.00	8.27	8.61	8.54	0.19
6	8.00	8.00	4.00	8.00	8.35	8.56	4.65	8.94
7	8.00	8.00	2.00	8.00	8.30	8.52	2.22	8.89
8	8.00	8.00	1.00	8.00	8.25	8.46	0.60	8.83
9	8.00	8.00	0.00	8.00	8.19	8.39	0.18	8.78
10	8.00	4.00	8.00	8.00	8.19	3.85	8.40	8.94
11	8.00	2.00	8.00	8.00	8.11	1.87	8.35	8.90
12	8.00	1.00	8.00	8.00	8.04	0.89	8.32	8.86
13	8.00	0.00	8.00	8.00	8.44	0.28	8.31	8.80
14	4.00	8.00	8.00	8.00	5.37	8.48	8.36	9.00
15	2.00	8.00	8.00	8.00	1.83	8.32	8.17	8.96
16	1.00	8.00	8.00	8.00	0.96	8.27	8.09	8.95
17	0.00	8.00	8.00	8.00	0.34	8.18	7.96	8.92
18	0.00	0.00	8.00	8.00	0.45	0.42	7.99	8.66
19	0.00	4.00	4.00	0.00	0.39	3.94	4.39	0.14
20	2.00	2.00	0.00	0.00	2.04	2.10	0.27	0.09
*R*^2^ of the network					0.996	0.998	0.997	0.999
